# Workarounds to Mandated Data Practices: Rethinking Digital Infrastructure in Child Welfare and Homeless-Serving Organizations

**DOI:** 10.1177/08912416251414048

**Published:** 2026-02-12

**Authors:** Aron L. Rosenberg, Naomi E. Nichols, Sarah Cullingham

**Affiliations:** 1 Research for Social Change Lab, Trent University, Peterborough, ON, Canada

**Keywords:** child welfare, data practices, discretion, homelessness, information management systems

## Abstract

Over the past decade, much of the infrastructure used for social service delivery in Canada has been digitized. Drawing on institutional ethnographic research practices, we undertook observational research, facilitated focus groups with twenty five key informants, and interviewed 121 leaders and workers in the child welfare and youth homelessness sectors to learn how data practices in these sectors are shaped by government-mandated infrastructure and tools associated with the adoption of digital information management systems in the public sphere. Despite promises that these new digital technologies will standardize and streamline data practices, we discovered that the introduction of digital information management technologies has led to a proliferation of ad hoc data practices, undertaken in local agencies to account for the shortcomings of government-mandated digital infrastructure. Our research suggests that the implementation of digital information management systems has not produced the promised efficiencies because they are incompatible with workers’ everyday social work practices, their service delivery aims, and their professional and ethical commitments. Because government-mandated data infrastructures fail to align with and support key aspects of people’s day-to-day work, social workers undertake a range of data workaround practices to fulfill their professional mandates. By focusing on these workaround practices, we pinpoint specific policy and infrastructural challenges facing social workers and point toward possible solutions to many of the problems they describe. Our institutional ethnographic approach helps us situate these practices within the broader relations organizing social service work, while the dual focus on child welfare and youth homelessness highlights how similar infrastructures generate distinct challenges across sectors. This cross-sectoral lens helps uncover both the limitations of one-size-fits-all digital reforms and the value of worker-centered processes for informing future infrastructure design.

## Introduction

Over the past decade, information and communications technologies (e.g., digital information management systems) have become a common part of the infrastructure in social service delivery throughout the Global North (Clarke 2020; Dobson 2022; Fink and Roholt 2022). In Canada and the USA, digital information infrastructure has been introduced with the promise of standardizing and streamlining the organization of data and data practices used in the child welfare and homelessness sectors (Grainger 2024; Marienfeldt 2024; Peressini and Engeland 2004; Willse 2008). In 2019, the Government of Canada launched Reaching Home – its homelessness funding program – mandating all municipalities utilize a homelessness information management system and employ a data-driven decision-making process to allocate scarce housing resources to the growing number of people who are homeless – presenting better data practices as part of the solution for homelessness (Infrastructure Canada 2023a). Similarly, in the Canadian province of Ontario, the Ministry of Children and Community Services requires all non-Indigenous children’s aid societies (CASs) to utilize a digital information management system (Government of Ontario 2023b) to streamline information sharing (Office of the Chief Coroner 2014) and improve accountability and efficiency (Government of Ontario 2024).

Despite all the attention and funding that has gone toward enabling the electronic turn in these fields, homelessness continues to affect more than 25,000–35,000 people in Canada daily (Strobel et al. 2021) with a 12% increase in homelessness since 2018 (Infrastructure Canada 2023b) and around 582,462 people annually in the USA – representing an increase of 6% since 2017 (National Alliance to End Homelessness 2023). Similarly, there are over 350,000 youth across Canada who are currently involved with child welfare services (Children’s Aid Foundation of Canada 2023) and there is no evidence to suggest that the use of digital information infrastructure in child welfare has improved in-care or post-care outcomes, nor is there evidence to suggest that fewer young people transition from care into homelessness. Homelessness and child protection issues persist despite the implementation of these techno-solutions. However, the introduction of new digital infrastructure has fundamentally shifted the day-to-day work of human service delivery. It is this shift, and in particular how it has unfolded from the standpoints of those delivering services, that is the focus of our research.

Informed by the mode of social inquiry Dorothy Smith (2005) described as institutional ethnography (IE), our research team has spent over two years speaking with people working in homeless-serving and child welfare organizations in Ontario, Canada, to learn about their data practices. Smith (2005) describes IE as an alternative approach to sociological research, rather than a methodology. Though interviews, text analysis, and observational research are common, institutional ethnographers use a range of methods and even distinctive methodologies in conducting their investigations (see Nichols et al. 2017; Nichols and Ruglis 2021). What binds this body of scholarship are intersecting ontological, epistemic, and ethical principles. Ontologically, IE upholds a materialist orientation to social life – meaning that our interest is in documenting people’s ordinary embodied activities (e.g., people’s work to conduct an investigation or clinical supervision in a child welfare context) as these connect to and shape the activities of people working elsewhere (e.g., youth shelter workers, parents), and how the co-ordered practices of individuals are shaped by institutional texts (i.e., policy, procedure, standards) and other human-made things, such as digital infrastructure, accounting technologies, or municipal infrastructure. Although research begins with people’s everyday experiences, the aim is not to generalize these experiences *as* findings nor as constituent of social theory; rather, an institutional ethnographer’s epistemic objective is to make generalizations about the institutional relations (i.e., procedural, discursive, infrastructural, technological, legislative, and policy) that contribute to patterns of social organization. These first two principles relate to the last. Ethically, an IE is meant to be useful to people, rather than generate or test theories *about* people. In this study, we were specifically interested in how people’s data practices – that is, their work to collect, input, access, review, analyze, store, and otherwise use data as part of their work – were shaped by new digital information management systems and data-driven workplace practices. We hoped our analysis would help people who work in the social service contexts understand why a set of reforms, designed to streamline their administrative lives, have resulted in a proliferation of new administrative practices.

This study asks, how do frontline social workers and managers conduct their data practices in response to government-mandated digital information systems? In IE terms, our study aims to explore how workers’ everyday data practices are organized by the ruling relations embedded in these digital information management systems and coordinated by the related technologies, practices, policies, and discourses. Existing research cannot fully answer these questions. While existing studies document workers’ use of discretion or workarounds in particular organizations, they seldom examine how these practices are organized by the broader institutional relations – policies, infrastructures, discourses, and accountability regimes – that span multiple sites. As such, they cannot explain why workaround practices emerge in different parts of the youth-serving system. Our research begins to fill this gap. Our approach is conversant with other scholars whose research seeks to help organizations “adapt to the constantly changing ecology of technologies – to improve their information management systems” (Voida 2014, 4) by conceiving of technological infrastructure as a shifting site of relations between ideas, technologies, and people. By anchoring our analysis of this context in ethnographic accounts of the ordinary data practices employed by social service workers working across two distinct but related sectors (child welfare and youth homelessness), our research pinpoints specific sociotechnical and policy conditions (e.g., database search functionality; audit and reporting requirements; organizational workflow) that constrain and enable workers’ practices, and which shape their orientation to social service modernization efforts. Our research extends what has previously been found about the significance of worker discretion (Busch and Henriksen 2018; Mathys et al. 2024; Mayer and Fischer 2023), arguing that workers’ everyday adaptations of and adjustments to government- or funder-mandated sociotechnical practices reveal essential insights regarding the efficacy and utility of digital information infrastructure for social work.

The article follows this outline. We begin with a review of the literature on institutional ethnographies of managerialism in public sector contexts, linking this to research tracing the adoption of new data practices associated with New Public Management (NPM) as both a governing rationality and a complementary set of technological shifts. We end our review of the literature with an exploration of the role that discretionary data practices play in relation to these governance reform initiatives. From here, we describe our own research design and analytic strategy, specifying how institutional ethnographers seek to make knowledge claims. We then present our ethnographic findings, describing the ordinary data practices of people working in child welfare and youth homelessness organizations and the generalized sociotechnical landscape that shapes their work. We follow that by explaining why this landscape and the related infrastructure require social workers to undertake additional data practices to work around technological limitations in order to realize the service delivery, reporting, and ethical objectives of their work. The article concludes with policy and practice recommendations, an overview of the limitations of our study, and directions for future investigations. We close with a discussion of the field, our approach to research, and the different organizational contexts of our work.

## Literature Review

Institutional ethnographers have produced a significant body of research that examines how people’s human service work has been reconfigured in relation to reforms associated with the NPM, that is, the adoption of “management ideas from business and private sector into the public services” (Haynes 2003, 9). While NPM is often associated with the introduction of market mechanisms into public service delivery, scholars have also noted that NPM is characterized by a broader managerial rationality emphasizing performance measurement, audit cultures, and standardized reporting practices (Haynes 2003; Griffith and Smith 2014; Lapuente and Van de Walle 2020). The digital reforms we examine in our research reflect this managerial strand more than marketization per se. We are interested in the intertwined managerial, accounting, and measurement regimes that continue to shape public and nonprofit work and are now being rearticulated through digital information systems.

IE scholarship has demonstrated the range of ways that NPM accounting and reporting technologies have reshaped the work practices of people in government (e.g., Ministries and government departments), public sector (e.g., publicly funded healthcare, schools, or child welfare), and nonprofit and charitable organizations (e.g., homeless shelters or food banks) – as well as changing relations between people working across these distinct but interrelated institutional contexts. For instance, *Under New Public Management* (Griffith and Smith 2014) is an edited collection of institutional ethnographies of changing frontline work practices across nongovernmental, nonprofit, and public sector contexts. The editors link these frontline shifts to a transnational program of reforms shaped by the globalization of capital and the growing influence of multinational corporations. As a collection, the volume demonstrates how a transnational economy translates into competition for multinational corporate investments and the concomitant pressure to lower corporate taxes and thus the costs of public service delivery (Griffith and Smith 2014). Griffith and Smith link these broader political-economic shifts to the adoption of managerial rationalities and practices across public and not-for-profit contexts (i.e., NPM) to create the economic efficiencies global market competition requires. This volume builds on the insights of earlier institutional ethnographies of managerial changes in public healthcare (Rankin and Campbell 2006) and public education (Kerr 2006; McCoy 1998; Nichols and Griffith 2009). Fewer IE studies have focused on how more recent intersections of NPM and digital reform efforts in these same contexts have once again shifted the grounds of people’s work practices and work knowledge within an organization and across sectors (see, for exception Corman 2017; Nichols et al. 2023; Nichols and McAuliffe 2024). Our research adds to the growing body of IE scholarship interested in the interpenetration of NPM discourses and practices and the calculative and surveilling technologies associated with the digital turn in human service delivery in Canada. Our research also contributes to the literature on governance in a digital era (Clarke 2020), public sector data practices (Ruppert 2012), and worker discretion (Raso 2017).

### Data Practices and the NPM

Writing about recent digital era public data governance in Canada, Clarke (2020) noted that in the last twenty years, a common orthodoxy regarding the digital era public management reform has emerged: “This common orthodoxy asserts that in order for civil service institutions to be resilient, effective, and relevant in a digital context, they must initiate radical [technological] reforms” (90). The digital turn in public sector governance has brought about significant changes to the way governments use technology like digital information infrastructure to meet contemporary expectations, address resource constraints, monitor and standardize practice, coordinate services, and so forth (e.g., Canada’s data-driven homelessness strategy [Government of Canada 2019b], Ontario’s child welfare redesign [Government of Ontario 2023a]). As governments lean into the digital shift, publicly funded arms-length organizations (e.g., child welfare agencies) and nonprofit organizations (e.g., shelters) contracted to provide social services are brought along for the ride.

In following the NPM transition, social service organizations are expected “to adapt to the evolving technical context” (Voida 2014, 6), embracing digital tools and systems that have become commonplace in business and private sector organizations – and, as our research demonstrates, have often been devised for these corporate contexts and then implemented in other settings. Before the NPM shift, “interest in data collection and understanding the performance of their programs was to further education. . .[or] used in demonstrating the conditions of the client population” (Mayer and Fischer 2023, 1). Although these motivations for data practices remain relevant in some social service organizations, data practices under NPM have become oriented toward “reporting to funders” (Benjamin et al. 2018, 185), including reports to government departments that contract nonprofit organizations to deliver services (Nichols 2014). Mathys et al. (2024) observe that these practices reflect and operationalize market-based rationalities in human service contexts: “the infusion of private sector ideas into the social service sector increasingly exposes social workers to the ideas of performance measurement and performance measurement instruments” (5).

Service-providing agencies are thus nudged to adopt the central goals of NPM – “efficiency and effectiveness” (Lapuente and Van de Walle 2020, 470) – which may not always translate into the best supports for all clients, particularly in complex situations where service efficacy may require *more* time and resources rather than less. Regardless, Mayer and Fischer (2023) show that “in pursuit of such effectiveness. . .some mix of performance measurement or program evaluation has become ubiquitous” (22). NPM “is founded on the idea that public [and subsequently, nonprofit] service performance can be strengthened by defining targets and outcomes. It advocates for continuous monitoring of these targets and outcomes” (Mathys et al. 2024, 6). But while the ubiquity of performance management frameworks across government, public sector, and nonprofit contexts has influenced the normalization of data practices in human service delivery, the tools and frameworks associated with NPM have not improved service efficacy nor efficiency.

Benjamin et al. (2018) found that NPM data processes, specifically associated with performance measurement and data-driven decision-making, do not support improved client experiences; furthermore, they found that available digital information infrastructure – largely designed to meet the needs of funders – is incompatible with organizational demands, including the need for organizational dexterity. Part of the problem is that the perceived link between efficiency and standardization has resulted in efforts to streamline social service practices through data-driven decision-making processes. Due to the “diversity and complexity” (Mathys et al. 2024, 7) of the people and needs served by social service organizations, standardized approaches are often insufficient. There thus arises a disjuncture between the standardized data-driven approaches that create efficiencies in an industrial context (for instance) and the fast-changing and relational demands associated with social service provision. In fact, writing specifically about child welfare and the way frontline workers use standardized risk assessment tools, Mathys et al. (2024) found that the promise of standardization associated with the use of structured decision-making and risk-assessment instruments “is more of an illusion than reality” (7), with different frontline workers coming to different conclusions when assessing the same young people with the same standardized tools. Our study contributes to the NPM literature by showing how digital reforms reshape frontline discretion while revealing how efficiency and standardization logics play out differently across organizational contexts (i.e., child welfare and homelessness services).

### Discretionary Data Practices

Mathys et al. (2024) address the challenges of standardized tools and processes by focusing on the “discretionary space” where frontline workers are required to make decisions based on “individual-level preferences and organizational-level variables” (5). Workers use discretionary space to creatively navigate standardized data practices while achieving other workplace goals. Mathys et al. (2024) found that “managers are likely to allow discretion and rely on the individual judgment and decisions of social workers” (9). Mayer and Fischer (2023) go further and insist on distributing these discretionary properties throughout an organization as a means to adapt standardized data practices to what they describe as “the heterogeneity in nonprofit data needs and uses” (7). Willermark et al. (2022) discuss this discretion as arising when “the digital tool at hand and the intended work task mismatch and force new practices to be created” (Willermark et al. 2022, 119). In these situations, “workarounds become a way of getting the job done” (Willermark et al. 2022, 118–9). While duplicate data practices, for example, may appear “irrational, creating more work for staff” (Benjamin et al. 2018, 201), they often conceal a disconnect between rigid data infrastructure and shifting programmatic and policy expectations that workers must also navigate. Information infrastructure is “modular, multi-layered,” operationalized via “social practices, norms, and individual behaviors” (Edwards et al. 2013, 5) and “shaped by an installed base of existing systems and practices” (Monteiro et al. 2013, 576). Digital information infrastructures thus both depend on, and constrain, people’s agentic practices.

By investigating people’s workaround practices – the myriad things they do to adapt, avoid, and/or manage the digital information technologies they must use to achieve their social service aims – this study adds to a growing body of knowledge on discretionary practices in social services, some of which from the same area where we conducted our research. For example, Raso (2017) interviewed workers serving people seeking welfare or “Ontario Works (OW),” as it is called in the province. Raso (2017) described how “caseworkers cleverly use discretion and adjust data entries so that these technologies produce decisions that are closer to workers’ interpretation of clients’ circumstances and OW’s legal regime” (78). More recently, Dobson (2022) wrote that “caseworkers attempting to bypass [the digital infrastructure used to administer OW]’s inflexible rules have discovered ways to subvert the system to help people who receive social assistance” (Dobson 2022, 60). Dobson (2022) even suggested that this inflexible infrastructure “almost forces the need for workarounds and learning ways to ‘game’ the system” (87). In this paper, we similarly focus on people’s pragmatic, subversive, and/or reparative data practices but in relation to Ontario’s child welfare and homelessness systems.

As we unpack below, many of our interviewees told us about their workaround practices as a way of illustrating how infrastructural limitations provoked them “to improvise and choose alternative ways to perform their tasks” (Willermark et al. 2022, 118). Informed by Dorothy Smith’s (1990, 1999, 2005) work on conceptual practices of power and the social organization of knowledge, our aim was not to identify examples of activities that helped us theorize the workaround as a specific manifestation of street-level bureaucracy (Lipsky 1980); rather, the concept directed our attention to the varied ways people created local adaptations and fixes that enabled their use of mandated technologies and to the sociotechnical landscape in which this happened. Our focus on people’s data practices – and especially their efforts to work around the limitations of the existing technological and communicative infrastructure – served as an opportunity to anchor our investigation of institutional discourse (e.g., that digitization and data-driven practice enhance efficiency and performance) and infrastructure (e.g., digital information management systems) in people’s actual work practices.

While prior institutional ethnographies have offered rich accounts of how digital infrastructures reorganize frontline work (Corman [2017] on paramedics, Raso [2017] on Ontario Works, and Benjamin [2019] on nonprofit data practices), much of this scholarship has tended to focus on single organizational settings. These studies highlight important dynamics of discretion, accountability, and resistance, but they leave relatively unexplored how distinct institutional arrangements – such as centralized provincial systems versus fragmented multi-funded service networks – similarly shape the uptake and consequences of mandated technologies. Our study addresses this gap by undertaking a dual-sector, cross-organizational analysis of child welfare and youth homelessness services in Ontario. By situating workers’ data practices within their organizational and institutional contexts, we show how technologies such as digital information management systems are not experienced uniformly but are actively shaped by the relational, material, and organizational conditions in which they are embedded. Conceptually, this extends IE analyses of managerialism and digital reform by foregrounding organizational setting as a constitutive factor in how technologies are enacted and adapted. Empirically, it opens new terrain by demonstrating how similar digital infrastructures generate connected tensions, workarounds, and discretionary practices across sectors. Our study thereby contributes to the NPM literature by showing how digital reforms are not simply layered onto existing managerial logics but actively reorganize the discretionary spaces of frontline workers, producing new forms of practice that both reflect and contest market-based rationalities. By including data from both child welfare and homelessness services, we demonstrate how the same policy logics of efficiency and standardization are mediated differently across organizational contexts, underscoring the limits of one-size-fits-all approaches to managing human services.

## Study Design

Our research focused on the interplay between people’s everyday activities and the digital, textually-mediated sequences of action that comprise work practices in the institutional and bureaucratic contexts of child welfare and homelessness services. The multisector design of our study is illustrative, not comparative. Our goal is not to compare child-welfare and homelessness, but to trace the translocal operation of digital infrastructure as it coordinates practices within both sectors. The heterogeneity of our sites demonstrates the reach and coherence of the institutional processes we are mapping.

### Methods

This research was conducted in two phases (Table 1). In Phase One we drew on key-informant interviews with sectoral leaders in government and academia (*n* = 12) and desk research to map the institutional data assets of the intersectoral youth-serving system that conditions young people’s lives from the moment they enter Ontario’s child welfare system (Nichols et al., 2023). To identify key informants (*n* = 12), we used a snowball sampling methodology by reaching out to key provincial coordinating bodies, including relevant government agencies. Key informant interviews were co-conducted via telephone, with one researcher asking questions while a second produced detailed fieldnotes that conveyed the content of each interview. These interviews were between thirty to sixty minutes in length. Using the map as a prompt, we then facilitated digital focus groups with key informants (*n* = 25) to gather their feedback regarding the accuracy and utility of the map we produced. Key informants, again recruited through a snowball sampling methodology, included technologists, lawyers, and policy analysts employed by the Ministry of Children, Community, and Social Services; social workers seconded to support the design and implementation of digital information management systems; child welfare researchers; and policy, technology, and research staff working in a non-governmental association of CASs. Sixty to ninety-minute focus groups were conducted using video-conferencing. They were audio-recorded and transcribed verbatim. We applied what we learned in these discussions to improve the map and produce a new version.

**Table 1.** Data Collection Summary.

Informants (Types and numbers)	Phase 1: (a) Twelve sector leaders in government and academia; (b) Twenty five technologists, lawyers, policy analysts, social workers, researchers, or association staff Phase 2: (c) 109 frontline and management staff
Duration	Phase 1: (a) interviews: Thirty to sixty minutes; (b) focus groups: Sixty to ninety minutes Phase 2: (c) interviews: Forty five to sixty minutes
Sectors and Organizations	Child Welfare: Two Children’s Aid Societies Youth Homelessness: multiple organizations across Ontario
Texts and digital tools analyzed	Policies and standards (legislation, program guidelines, funding agreements, reporting requirements, government communications) Organizational documents (training materials, standards, internal guidelines) Digital tools (information management systems, reporting tools, local files, dashboards)

In Phase Two, we worked with two CASs and youth homelessness organizations across the province of Ontario to dig deeper into some of the issues raised during the first phase of the project. One of the CAS agencies was identified in Phase One when the Director of IT and quality assurance participated in a key informant interview. He invited our Prinicipal Investigator (PI), Dr. Naomi Nichols, to present the study to the directors of the agency, who elected to participate on the basis of this presentation. After presenting the map to people who work in quality assurance across the province of Ontario, another CAS agency contacted the PI about participating in the study. Again, a presentation to the directors led to the formal collaboration. To recruit participants that work in youth homelessness sector organizations (organizations that are much smaller and less complex than a child welfare agency), we gave a research presentation of Phase One findings to a knowledge exchange network and utilized the network associated with one of our funders (Making the Shift) to solicit other participating individuals and one large multi-service organization, where the majority of our observational research was conducted.

On the basis of our recruitment efforts, we completed forty five to sixty minutes in-depth interviews with 109 frontline and management staff in two child welfare agencies (one large urban setting and one smaller rural/urban setting); as well as people working in youth homelessness and related organizations across the province, but especially in one large urban setting in Ontario. Interviews with child welfare workers were conducted using video-conferencing software, audio-recorded, and transcribed verbatim. Interviews conducted with people who work in the youth homelessness sector were conducted face-to-face or using video-conferencing software – all were audio-recorded and transcribed verbatim. We employed a co-interviewing protocol to enable extensive notetaking during interviews, allowing us to keep track of emerging insights, topics, and questions. The interviews focused on people’s data practices and the ways these are shaped by digital infrastructures and tools (e.g., specific information management systems). Text and document analyses began during Phase One and has continued throughout data collection.

As an example of text and document analyses, when references to an organizational or policy text were made during interviews, we looked them up online or asked for copies to review. We employed Smith’s (1990, 2005) broad conceptualization of texts – that is, replicable artifacts that coordinate people’s practices across time and space – in our study, and investigated the role digital artifacts play as a kind of coordinating text in social service work. Our focus on digital artifacts was also informed by Corman (2017)’s institutional ethnography of digital technologies used by paramedics. To understand how everyday digital technologies are used in practice, we conducted digital observations with people who work in IT and quality assurance for the province, asking for demonstrations and tours of relevant programs or technologies to improve our understanding of their functionality and utility. We also participated in free online training about the digital tools we were told about, and gathered and reviewed training slide-decks and materials mentioned by key informants. Members of our team (including co-author, Sarah Cullingham) also conducted five days of observational research in youth homelessness sector organizations across a single municipal context. The observational research included daily job shadowing and informal conversations, with a specific focus on how people are engaging with specific technologies and infrastructure as part of their everyday work. For example, we invited people to show us how they use a particular technology in their interactions with youth (e.g., a frontline worker showed us what information is put into the federal homelessness information management system and what information is recorded in the local database, explaining how these decisions are made). Fieldnotes were produced to document our evolving understanding of social welfare data practices. The combined use of textual, observational, and interview analyses has allowed us to identify some of the underlying structural (i.e., policy, technological, procedural, discursive) factors impacting people’s work.

### Analytical Approach

In institutional ethnography, the identification of common experiences – patterns in how people talk about their work – is the initial step in analysis. To produce the first level of analysis, we developed and utilized a code-book that allowed us to systematically index interviews to identify the range of data practices people employ, the types of data they generate or use, as well as the infrastructural, legislative, procedural, instrumental (e.g., assessment tools), discursive, economic, and other institutional relations that condition the data practices people described. The codebook was developed by the research team, under the mentorship of the PI (Nichols), by reviewing the original study design and several transcripts selected to ensure geographic, institutional, and professional representation. All transcripts were coded by one research assistant and checked by another, including the PI. The team met weekly during coding to review and discuss progress. Based on the first phase of analysis, several common challenges faced by workers in the homelessness and child welfare sectors were identified. When disagreements in interpretation emerged, the team returned to the transcript and its surrounding context, collectively re-reading and discussing it until we arrived at a shared, context-grounded interpretation that aligned with IE’s commitment to preserving the integrity of informants’ accounts.

From there in IE studies, researchers seek to explicate the textually-mediated relations shaping the patterns that have been identified (Smith 2005). Coded data point toward specific texts, technologies, discourses, and infrastructure that are used by workers. To produce the second phase of analysis, the research team brought together the interview, observational, and text data to produce an empirical explanation for the data challenges people described in the interviews. The intention is not to generalize people’s experiences as instances of a theme or a concept (in this case, workarounds); rather, we are trying to explain how the experiences people describe are organized by generalized institutional relations. Ethnographically, we focus on documenting common text-act-text-act sequences of action. Our data processing and analytic practices thus seek to bring into focus the institutional contexts shaping people’s work – as is emblematic of institutional ethnographic ways of working with data (DeVault and McCoy 2006).

Our study is not an ethnography in the conventional sense of extended immersion in a single field site. Rather, it follows the methodological commitments of IE, which begins from people’s everyday experiences but traces how those activities are coordinated by extra-local texts, technologies, and ruling relations (Smith 2005). IE does not require prolonged participant observation within a bounded setting; instead, it relies on interviews, textual analysis, and strategic observations to map the social organization of work across sites. Our data collection – interviews with workers and managers across multiple organizations, textual and technological analyses, and targeted observations – aligns with this mode of inquiry, which aims to illuminate the institutional relations shaping people’s activities rather than produce a holistic account of a single field site.

## Findings

Almost unanimously, the people we interviewed expressed frustration that the digital information management technologies they were required to use in their workplaces did not allow them to efficiently and effectively achieve their professional objectives – despite governing narratives which position data-driven practices and new digital infrastructures as the way to improve efforts to address complex social problems. Summing up this collective sense of frustration, Bettie, a senior manager of child welfare services exclaimed during an interview: “The words that drive me crazy since 2015 when this [the Child Protection Information Network or CPIN] was implemented, ‘workarounds’. . . Don’t give me another workaround, man!” The analysis offered here seeks to understand how the implementation of digital information management and data-driven decision-making tools, ostensibly designed to streamline and standardize social work practices to improve the efficacy and visibility of people’s work, have resulted in the proliferation of workarounds – that is, local adaptations of provincially or federally managed infrastructure and tools.

Workers we spoke with described the mandated digital infrastructure central to their work as time-consuming, resource-intensive, and yet to deliver on their promises of improving services or client outcomes. As one frontline worker explained, “you’re just constantly clicking, constantly repeating yourself. And important information isn’t easy to find.” Our inquiry begins with the tension between the promise that government-mandated information management systems will standardize, streamline, and improve social work, and people’s experiences that this infrastructure actually multiplies their administrative tasks, limiting their potential to support clients. By focusing on all the ways that workers continue to use discretion to make up for the shortcomings of the imposed technologies, our analysis reveals precisely how new information infrastructure fails to actualize the promises of data-driven practice and digital information management technologies. People cannot meaningfully use the information infrastructure available to them to achieve their social welfare aims – for example, keeping kids safe and supporting their wellbeing or getting people housed – at least not without exercising their agency through cumbersome workaround practices. In this way, we move beyond a theorizing of workarounds toward an ethnographic account that shows how particular mandated digital information infrastructure obstructs and complicates people’s social welfare work. We begin by laying out the sociotechnical terrain upon which people in the youth homelessness and child welfare sectors are carrying out their work.

### Child Welfare and Youth Homelessness: The Sociotechnical and Policy Backdrop

The Ontario and Canadian governments have been embracing the digital era in public service delivery, as evidenced by “Ontario’s recently introduced *Simpler, Faster, Better Services Act* (2019), and in the introduction of new senior leadership roles across a range of Canadian governments” (Clarke 2020, 101). In this context, information management systems are expected to enable the collection, storage, analysis, and sharing of data between departments, organizations, or various levels of government. As part of Canada’s 2019 federal homelessness strategy, organizations that receive government funding are required to adopt a “Coordinated Access” approach to service delivery that must include a Homeless Management Information System or HMIS to coordinate and standardize worker practices (Government of Canada 2019a). An HMIS changes not just how case notes are recorded, but what can be recorded, and in what forms” (Willse 2008, 231) – suggesting, as Ruppert (2012) notes, that databases have ontological effects.

In Canada, the most common HMIS is the Homeless Individuals and Families Information System (HIFIS), which was developed by the federal government and is provided freely to homeless-serving organizations. HIFIS is meant to promote collaboration among agencies, streamline intake processes, and support resource allocation decisions; it is also expected to provide aggregate, but anonymized, homelessness demographics to senior management and government (Government of Canada 2019b). Unfortunately, as Benjamin et al. (2018) observe and as our own research affirms, it can be challenging for one HMIS to serve so many different functions and parties. The kinds of data that might be helpful for the internal workings of an organization are often different from what government, funders, or other external stakeholders might be interested in, and HMIS systems can rarely support everyone’s needs.

Similar to HIFIS, the Child Protection Information Network (CPIN) is a digital information management infrastructure utilized for social service provision in Ontario’s child welfare sector. It was introduced after a public inquiry about a child’s death to help standardize decision-making and reporting while ensuring all of Ontario’s CASs have access to the same information about a child or family (Ontario Association of Children’s Aid Societies 2016). CPIN centralizes case management, information sharing, and reporting (Government of Ontario 2015) and aims to improve data accuracy, enhance service coordination, and facilitate timely decision-making in child protection cases (Government of Ontario 2022). CPIN has been used since 2014 and has faced major issues throughout its development and deployment, many of which persist today (Clarke et al. 2018; Mielniczuk 2014; Nichols et al. 2023; Nichols and McAuliffe 2024).

For the participants in our study, the imperative to engage in the specific information infrastructures described above comes directly from government and serves as a means of operationalizing legislative commitments, demonstrating compliance with federal directives or provincial standards, and performing economic accountability. However, our research also uncovered other influences at play – such as organizational values with respect to client confidentiality, workflow issues related to data collection and access in the field, and concerns with quality assurance – that necessitated other data practices than those imposed by the state. As our interviewees explained, all of these other data practices, or workarounds, involved a reliance on (a) manual and interpersonal alternatives to automated digital processes (e.g., manual file crawls, having conversations, writing in notebooks, Excel, or Word, etc.), (b) data double-up practices (e.g., using secondary databases), and (c) bespoke programs (e.g., alternative financial reporting models and customized dashboards).

#### Manual and Interpersonal Workaround Practices

As new digital tools and systems have become mandated for use in child welfare and homeless-serving organizations, workers we spoke to often expressed surprise at how many of their data practices must still be completed manually and/or interpersonally, despite the supposed automating affordances of a digital infrastructure. Regardless of government claims that information management technologies improve efficiencies and enable streamlined work organization, interviews with frontline workers, supervisors, and senior management offered many examples of ways in which these new practices were incompatible with service delivery aims and timelines (or simply ineffective), leading people to rely on time-consuming manual data practices in the youth homelessness and child welfare sectors.

##### Child Welfare Services and CPIN

A frontline worker, Marge, told us about her experiences trying to generate what she described as her weekly “personal report card” – more formally called a Cognos report. The Cognos report is meant to synthesize relevant CPIN data to help frontline staff identify outstanding tasks that must be completed for a worker to be compliant with agency and government regulations. The idea is to make compliance monitoring more efficient, but many of those we interviewed complained that the Cognos reports are not user-friendly and did not necessarily enable accurate compliance monitoring. For instance, Marge noted that her reports were inaccurate: “no matter how many home visits I have, I’m 1142 days behind on my home visits [in the Cognos report]. I don’t know why. I check all the right boxes [in CPIN]. I document everything properly. [Cognos] just doesn’t ever read it.” This technical glitch means that Marge’s supervisory team view her progress as non-compliant. To protect herself and her job, Marge continually reviews her files manually to make sure she has accurate information to defend herself despite what her Cognos reports indicate.

Cognos is the only tool most workers have for extracting aggregate information from CPIN, but its functionality is limited. It was not designed for agency-level use; rather, its standardized features were primarily developed to allow the provincial government to monitor worker and agency compliance with the Child Protection Standards. Cognos was originally developed and deployed by IBM, although the government of Ontario now employs their own workers to maintain and update the tool. IBM (2022)’s website claims that, with Cognos, “you can create any reports that your organization requires.” However, this promise is not always borne out in practice. Cognos is largely used to export compliance-related data from CPIN. If people want to extract other types of information, these reports are often completed manually, depending on available local IT resources (e.g., while large child welfare agencies may have an IT department, smaller agencies often only have a single quality assurance staff). For example, Sophie, a supervisor, recalled being unable to extract the necessary information from CPIN using the Cognos reporting tool when she was asked about “the number of access visits that were scheduled versus the number that families actually attended.” Because there is no standardized Cognos report to aggregate and extract this information from CPIN, Sophie or someone on her team had “to go through and actually look at each of the contact logs because [they] don’t have a method to track it beyond that.” Sophie described this manual workaround as “a tedious task because if a family’s been having access, you know, three days a week for eight months and now suddenly were us to pull this information, it is time consuming.” Sophie’s data practices point toward a digital information management system that enables Ministry oversight of arms-length agencies but is not terribly useful to social workers nor the managerial staff tasked with monitoring social caseloads.

Although CPIN continues to receive Ministry updates, it does not enable the types of nuanced analytic practices that social workers believe would advance clinical practice. For instance, Lucy, another supervisor, explained that although a Cognos report can capture how many files each worker has, it is not able to differentiate case complexity or the amount of work each file might represent. To work around these limitations, she – as a supervisor – still manually goes through each of her workers’ files:

I’ll go through it to understand, Yes, that person has seventeen files, but I know those files are like, there’s a permanency plan they are going to close in the next month. So, it helps me that way, too.

This manual review provides Lucy with a more accurate understanding of her team’s workloads than what she can discern from Cognos’ standardized reporting features alone. Cognos reports assume commensurability between individual cases and have been designed to enable monitoring of the completion of specific time-based activities (associated with the implementation of the Child Protection Standards). Manual file reviews and meetings with workers allow supervisors like Lucy to understand and monitor qualitative differences between the caseloads of those under their supervision.

Manual processes are also often preferred by frontline workers. A third supervisor, Louise, explained that most of her workers “have their own like, Word documents or Excel documents that they keep track of, or just paper and pen, like [laughter] everybody’s different. . .I also have my phone for reminders of different things.” Because CPIN data are warehoused off-site, the manual workaround practices ensure pertinent information is close-at-hand and easily accessible from the field. Local alternatives are often experienced as more straightforward or easier to access than extracting back daily Cognos reports from CPIN. Among managers and frontline workers, practical requirements for accessible and accurate information in the field and/or in a timely and usable manner at the office requires the creation of low-technology workarounds for information generation, workflow tracking, and information management.

##### Homelessness Services and HIFIS

People’s information management practices in homeless-serving organizations differ from those in child welfare, but a common thread is that – in both contexts – the data stored and managed by provincial (child welfare) or federal (homelessness) infrastructure is insufficiently usable at the agency or organizational-level to adequately enable case management, quality oversight, or assessments of service outcomes. A central difference between the two contexts is that while CPIN and its associated tools are explicitly oriented to assessing legislative compliance and institutional accountability, in the homelessness sector, people described being unable to use the existing infrastructure even to enable basic accountability work with funders and government. In part, this is because – unlike provincially mandated child welfare agencies – homelessness organizations have multiple funders, including various provincial and federal ministries, municipal governments, philanthropic foundations, and private donors – all of whom require different reporting standards.

In homeless-serving organizations, although information management systems must be used by organizations that receive federal funding, not all these organizations can access the data they enter into these HMIS systems. For example, as the data administrator for her municipal government, Elana has the authority and capacity to use the data in HIFIS, but many of the direct service organizations she works with do not. They must therefore send their data to Elana who adds it on their behalf:

So, for the organizations that don’t have direct access to HIFIS. . .whether that be for the reason that we just haven’t trained them, or they don’t think they would use it enough to warrant putting their staff through that kind of training, they provide me the information on paper. And then either myself or one of the admin assistants that help me out. . .manually type it in and put it in the database. And so that information is provided to me using a secure online form. I tell people not to email it to me and not to fax it to me because there’s lots of room for inappropriate disclosure, accidental disclosure by sending information that way.

Here, Elena describes a sociotechnical landscape where organizations do not have the training, technical capacity, or permissions to meaningfully and fully interact with the systems they are mandated to use. Unfortunately, this means that the workaround that Elena describes above (i.e., manually providing her with a hard copy of the data to add manually to the database) risks the very privacy concerns that access to a protected database is meant to alleviate. The lack of access faced by some organizations also means they cannot use HIFIS to guide their social welfare practices.

The issues with HIFIS become even more pronounced when we spoke with people who have more specialized roles in agencies serving homeless youth, and who are expected to communicate program results and other information to multiple funders. Essie, for example, who runs a substance-use program at a homeless shelter for youth, is required to provide funders with regular updates about client and assessment volume but neither of the information management systems that she uses (HIFIS), nor another digital case-management software used by the agency (EMHware), provide the functionality she requires to complete her reports to funders. So instead, she relies on spreadsheets and Word documents:

I create a big spreadsheet for the program manager, counting how many unique clients accessed for the month, how many assessments are getting done every night. We don’t have a spot really to track that on EMHware, or on HIFIS, but we have a Word document where we are tracking how many assessments are done because that shows a lot of the work that gets done that kind of gets missed [in the information management systems]. . . So, it gets reported to the city [who requires the organization to use HIFIS], but through us rather than like HIFIS or anything like that. . . Because the city wants that information, [laughter] but they haven’t provided a way to give it.

In her ideal world, Essie wishes the data management system (HIFIS and/or EMHware) could generate reports for her, consolidating information so that she would not have to spend the time manually going through case notes and preparing spreadsheets.

##### Cross-Sectoral Commonalities

Indeed, the lack of priority granted to system usability for participating agencies is a problem common to both the child welfare and homelessness systems. From our interviews with senior provincial managers in the child welfare system, we learned that only 14% of workers’ requests to improve CPIN’s ability to support client well-being or service quality have been instituted since CPIN’s inception. Programmers are employed by the Ministry (not individual agencies); as such, they are required to prioritize requests by government to align the information management system with legislative changes, compliance and accountability goals. Agency-requests for improvements to local functionality are not prioritized.

#### Pragmatic and Ethical Data Double-Up Practices

As we described, people often rely on additional data practices, undertaken using Microsoft Excel, Word, or even analog options like a pen and paper, in addition to their mandated use of CPIN, HIFIS. In child welfare, for example, beyond just pulling Cognos reports from CPIN, Ellie, a child welfare supervisor, uses an Excel spreadsheet that she stores on local shared drives. She explained that certain “internal documents” are not compatible with CPIN, so “they’re housed just on [a local] server.” Similarly, in homeless-serving organizations, additional programs – like EMHware – are used in addition to HIFIS because these software, designed specifically for case management, are more useful and usable in client-serving organizations. Furthermore, like with CPIN, concerns about the reliability of HIFIS lead people to implement local data back-up strategies. Chip, a lead youth support worker, explained that his referral numbers were not being accurately tabulated by HIFIS, so management at the agency suggested they “double up” their data practices and use EMHware too and then “compare the numbers and see.” Others see their data duplication practices as way to keep sensitive client information out of shared information management systems, like in this quote from Essie:

HIFIS is also visible to all the other shelters in [town]. Sometimes we don’t always include things. . .There is the ability to do things like case notes on HIFIS and enter in a lot more information, but we don’t because other shelters can view it . . . [if, for example, we include] our incident report that this youth [was] discharged for fighting someone outside, that shelter can view that. And they might say, “well, we don’t want to take that person, because we know that they were discharged because they got in a fight”. . . So, we’re kind of strictly use it for inputting the services we’re providing, goods and services, referrals, housing. . . Whereas our actual case management is all done through EMHware.

In this example, Essie’s doubling-up practices ensure that local case-management information cannot be used to restrict youth from receiving services elsewhere. Shelter workers must record goods and service utilization in HIFIS as a requirement of their service contracts with municipal governments; this requirement contours their work with people seeking shelter, but it does not determine it. Rather, in Essie’s account, doubling up data practices comes across as a subversive attempt to actualize shared organizational values around client confidentiality and collective local work without compromising the terms of their service contracts with the municipality.

#### Developing Bespoke Programs

In some circumstances, funder- or government-imposed information management systems were so incompatible with agency needs that workers and organizations have developed bespoke programs and customized technologies to make the mandated data infrastructure usable. For example, Jay, a CAS financial manager, explained that trying to complete his financial data practices using CPIN has been “more time consuming and definitely not as useful” as with the previous information management system used at his agency. One of the stated justifications for adopting CPIN was better financial management and oversight (Government of Ontario 2023b). However, like the challenges frontline workers face using Cognos reports to export data warehoused in CPIN back to the agencies, Jay observed that CPIN does not have the capacity to generate useful financial statements without time-consuming manual workaround practices:

With CPIN, basically, all it is, you get the raw data, and then you have to sort of create, you know, the reports or template yourself. . .so there’s an extra amount of work involved, each and every month to get the information out of CPIN and put it in a format that’s useful.

To help with this extra work, Jay developed a program to process CPIN’s financial data into useful formats.

The other bespoke solution we will describe – a unique digital program developed to make CPIN’s data usable for local agency needs – is the dashboard that some CASs use to review, aggregate, and analyze data. Because the government does not allow CASs to store their files locally – for security reasons – agencies sometimes create or adopt a program to download several data sets daily and upload them into an agency’s local database (called their “dashboard”) in ways that aim to support service delivery. Not all CASs have a dashboard. However, Hameed – one of the senior managers with a larger CAS that does have one – gave us a demonstration of the program. As Hameed showed us, the custom-built dashboard provides their agency with more access to, and control over, the data they gather and use. However, even with this dashboard, a lack of analytic capacity within the agency (that is, people to engage with this data) means the tool is still not realizing its potential of supporting more local engagement with the information in CPIN. The promises of data-driven service provision that CPIN was developed to facilitate (the ability to use data to improve client outcomes) remain constrained by the mandated technological infrastructure as well as a persistent lack of fit between the dynamic and relational realities of social service practices and the narrowly standardized requirements needed to ensure CPIN can be used to generate meaningful information.

## Discussions and Conclusions

Although the use of digital information management technologies is expected to homogenize and improve workers’ practices and clients’ experiences (Government of Canada 2022), our research suggests that new government-mandated infrastructure does not improve people’s capacities to actualize professional objectives (e.g., protect children; end homelessness) nor has it streamlined communication and reporting practices. Technological advancements are often presented as silver bullets to complex social problems (Joyce et al. 2023; Rosenberg et al. 2023). Our research uncovers ways in which technological innovations are failing to coalesce helpfully with social workers’ and clients’ needs.

Primarily, we found that digital infrastructure developed to enable external monitoring of legislative compliance, when coupled with the adaptation of digital tools developed for corporate-reporting practices, fundamentally fail to enable the types of data practices that frontline workers need and want to do in order to fulfill their professional mandates. For example, file sharing and use is obstructed by the complexities and limitations of the information management systems that are not accessible during field-based clinical work (e.g., in child protection investigations); this infrastructure does not enable the case management, reporting, nor financial management that is required of people. Beyond issues with the technological tools themselves, human resource challenges limit the utility of these systems: some agencies and staff lack the training or access they might need, accurate aggregated data is difficult for people to compile or review, and workers end up spending as much – or more – time fulfilling the mandated data practices to protect their job as they do fulfilling their primary duty protecting their clients.

By investigating the data practices workers have devised to meet their clients’ needs and fulfill mandated data practices, we found that – in its current form – digital information infrastructure actually obstructs people’s potential to realize effective, efficient, and ethical service provision; these infrastructure (1) lack the capacity to support diverse and nuanced frontline data practices – especially for workers who spend much of their day outside of the office working directly with children, youth and families, (2) result in a proliferation of additional data practices, rather than streamlining information management and communications procedures within and across agencies, and (3) jeopardize client privacy or reputation and potentially limit access to life-sustaining services (e.g., emergency shelter beds). As people told us about their strategies for complying – or attempting to appear compliant – with technical and reporting requirements while also upholding their service commitments, it became clear that people’s use of new digital information management systems enables a performance of standardization; all the while, people continue to rely on cumbersome local adaptations or “workarounds” to carry out their social service duties. Our work uncovered the dissonance between the promise of efficiency and standardization associated with the implementation of digital information management systems and the reality that people’s data practices have proliferated to account for the shortcomings of government-mandated infrastructure and tools.

### Recommendations

An alternative approach to infrastructure development might build out these supports from the ground up – that is, by embedding computer engineers and data scientists in the local settings where the infrastructures are meant to be deployed or by involving social workers from these local settings in the technological development of digital infrastructures in more meaningful ways. Such processes would treat frontline staff and other local stakeholders not as end-users to be trained, but as co-designers with critical insights into how technologies are actually mobilized in practice. This requires ongoing opportunities for staff to shape and revise infrastructures, ensuring that systems evolve with the complexities of service delivery.

Practically speaking, child protection workers would benefit from data practices and associated infrastructure that accommodate the generation and use of information in the field – that is, during investigations or visits to children, youth, and families. The lack of timely access and limited functionality associated with the process of extracting data back to agencies undermines government claims that modernization creates local efficiencies. Relatedly, reporting tools developed for the business sector (e.g., Cognos reports), adapted for government oversight of legislative compliance, and imposed in social service contexts, like child welfare, do not enable streamlined nor timely engagement with evidence to support service enhancements. Social service workers employed in the youth homelessness sector, on the other hand, would benefit from information management infrastructure that can accommodate and support their multiple reporting realities. This is particularly true for agencies providing a range of mental health, addiction, social, educational, etc. – as well as housing – supports for the young people they serve. Ideally, information infrastructure would support information sharing, program assessment, learning within agencies, and outward communication to an agency’s multiple funders – without unnecessarily exposing young people to government surveillance. Infrastructure development that does not consider the wider sociotechnical relations comprising human service delivery is not viewed as useful by those who are required to use it. As such, they initiate or continue with data practices and infrastructures that *do* advance their workplace aims.

### Limitations and Directions for Future Research

While our study provides a detailed institutional ethnographic account of data practices in Ontario’s child welfare and youth homelessness sectors, there are limitations to note. First, our data was drawn from a single provincial context, and while the findings resonate with broader shifts across the Global North, the specific institutional arrangements of Ontario’s public sector shape how technologies are taken up locally. Second, although we interviewed a wide range of workers, from frontline to management, our focus remained on staff perspectives rather than client experiences of digital infrastructures.

Future research should extend this dual-sector analysis to other jurisdictions to examine how different governance models and policy environments shape the uptake of digital infrastructures. Additionally, further research could incorporate the perspectives of clients, who experience both the benefits and harms of how information infrastructures mediate access to services and privacy. Finally, studies that follow the development cycle of digital systems could help chart pathways toward more participatory, worker-centered design processes.

### Humans in the Machine

By showing how workers’ so-called workarounds are not merely idiosyncratic but systematically provoked by institutional arrangements, IE makes visible the ruling relations that shape practice. This reframing helps workers, organizations, and policymakers see that the problem is not frontline resistance or incompetence, but the misalignment between institutional data regimes and the lived requirements of service delivery. In this sense, IE helps people understand the mismatch in a new way: not as a failure to implement technology correctly, but as evidence of how institutional demands and frontline realities are organized through competing logics – one oriented to accountability and audit, the other to care and responsiveness.

Because IE attends to how work is socially and institutionally organized, we also considered the specific organizational contexts in which data practices unfolded. Child welfare agencies, with their centralized compliance and accountability structures, created different technological pressures than youth homelessness organizations, which operated in more fragmented funding environments with multiple reporting requirements. These organizational contexts – marked by different governance arrangements, resource constraints, and professional mandates – created distinct pressures that conditioned how workers interacted with digital infrastructures, when they resorted to workarounds, and what forms those workarounds took. Situating data practices within these organizational settings highlights how institutional contexts do not merely provide a backdrop to technological use, but actively configure the very possibilities and limits of how technologies are experienced in practice.

Through a sociotechnical lens, workers’ frustrations with new technologies that do not meet their professional needs serve as a reminder that digital information infrastructure can only take us so far. While systems meant to support homeless people and the children or families involved with child welfare continue operating without sufficient resources or services, the potential benefits of digital innovations cannot overcome this scarcity. As a senior project manager with homelessness services told us, “we can be as integrated and sort of smooth in our processes but if the housing is not there, it’s not going to make any difference.” In fact, the time-consuming and cumbersome data practices we describe in this paper may be further entrenching the scarcity that justified digital innovations in the first place.
